# No Evidence for Male Retaliation in a Population With High Level of Extra‐Pair Paternity

**DOI:** 10.1002/ece3.71423

**Published:** 2025-05-07

**Authors:** Helga Gyarmathy, Renáta Kopena, Tünde Kneifel, Fanni Sarkadi, Eszter Szöllősi, Eszter Szász, János Török, Balázs Rosivall

**Affiliations:** ^1^ Behavioural Ecology Group, Department of Systematic Zoology and Ecology ELTE Eötvös Loránd University Budapest Hungary; ^2^ Doctoral School of Biology, Institute of Biology ELTE Eötvös Loránd University, Institute of Biology Budapest Hungary; ^3^ Evolutionary Ecology Research Group, Institute of Ecology and Botany HUN‐REN Centre for Ecological Research Vácrátót Hungary; ^4^ Department of Reference Sample Analysis Institute of Forensic Genetics, Hungarian Institute for Forensic Sciences (HIFS) Budapest Hungary; ^5^ HUN‐REN–ELTE–MTM Integrative Ecology Research Group ELTE Eötvös Loránd University Budapest Hungary

**Keywords:** certainty of paternity, cuckoldry, extra‐pair mating, feeding frequency, mating cost, paternal investment

## Abstract

Extra‐pair paternity (EPP) is a widespread phenomenon, as EPP has been observed in 76% of the socially monogamous bird species. Many hypotheses try to explain the evolution of infidelity. While females may participate in extra‐pair copulations, for instance, to ensure the fertilisation of their eggs or to obtain potential genetic benefits for their offspring, unfaithful females face many potential costs too. As nestling provisioning is one of, if not the most energetically costly forms of parental care, the certainty of paternity hypothesis predicts that males with an unfaithful partner reduce their parental investment to avoid the fitness loss arising from rearing unrelated nestlings. We investigated the relationship between the presence and proportion of extra‐pair young (EPY) and the feeding rate of the social male to reveal whether males recognise and penalise unfaithfulness. We conducted the study in a Hungarian population of collared flycatchers ( *Ficedula albicollis* ) where the EPP rate had been reported to be high. We cross‐fostered nestlings so that each parent reared offspring from two foreign broods and none from their own. Thus, any relationship between paternal investment and paternity in the original brood of the male should be the direct consequence of the female's mating behaviour (as perceived by the male) and not the result of early maternal effects or different behaviour of extra‐ and within‐pair offspring. We found that 63.6% of the broods contained EPY, and 23% of the nestlings were sired by extra‐pair fathers. The only relationship we found was that males with larger broods fed their offspring more frequently. Neither the prevalence nor the proportion of EPY was related to the male feeding rate; thus, our results do not support the certainty of paternity hypothesis. This might be explained by the inability of the males to track their females' behaviour in a population with a high EPP rate.

## Graphical abstract

In an experimental study, we investigated the relationship between within‐pair paternity of the males and their feeding rates in a monogamous passerine with a high rate of infidelity to reveal whether males recognise and penalise the unfaithfulness of their social partners. Our results contradict those previously found in another population of the same species. We argue that the contradictory findings may be explained by the dissimilar rate of extra‐pair copulations in the two populations and the trade‐off between males' extra‐pair mating activity and ability to assess their within‐pair paternity reliably.

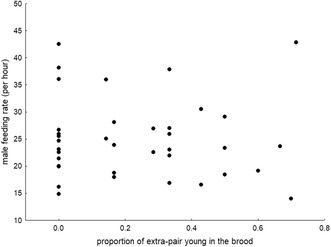

## 1 Introduction

From the late 80s, extra‐pair paternity (EPP) has been observed in a large number of species (Brouwer and Griffith 2019; Lifjeld et al. 2019). In their study, Brouwer and Griffith (2019) reported that fertilisation outside of the social pair bond was found in 76% of the surveyed socially monogamous bird species. Species show high variation in the proportion of broods containing extra‐pair young (EPY) even within families (e.g., Paridae, Hirundinidae; see Table S1 in Brouwer and Griffith (2019)). In some species, considerable variation exists also among populations. For instance, in a Norwegian population of great tits ( *Parus major* ), EPY were present in 27.3% of the broods (Krokene et al. 1998), while in a Spanish population, this rate was as high as 55.3% (García‐Navas et al. 2015). These results highlight the importance of considering both the social and the genetic mating systems of a species when conducting behavioural studies and raise many questions regarding the evolutionary drivers of infidelity.

Considering the males' perspective, extra‐pair copulations (EPCs) can increase the number of nestlings so that the males do not have to pay the costs of rearing the surplus offspring. As the number of eggs a female can lay during a breeding event is limited (Monaghan and Nager 1997), the benefits are less apparent for females. Still, numerous hypotheses have been proposed to explain the motives behind female infidelity (reviewed in Brouwer and Griffith 2019). Extra‐pair mating may provide insurance against the infertility of the social partner (e.g., Santema et al. 2020; Wetton and Parkin 1991), and/or offer potential indirect genetic benefits. For example, females may enhance the genetic quality of their offspring through “good genes” (Hasselquist et al. 1996) or increased heterozygosity (e.g., Foerster et al. 2003; Stapleton et al. 2007). EPCs may also bring direct benefits for the females when extra‐pair males offer courtship food, protection against predators, or contribution to parental care (reviewed by Brouwer and Griffith 2019), albeit the direct benefits are relatively poorly investigated (see in Santema and Kempenaers 2021).

Although some studies have provided supportive results for some of the adaptive hypotheses (see e.g., the references above), a general support is missing. In their recent meta‐analysis, Hsu et al. (2015) found no difference in size or genetic similarity between the extra‐pair male and the social partner of the females (but see Arct et al. 2015 for an opposing result on genetic similarity and Reid 2015 for a cautionary note). On the other hand, there was an overall nonsignificant trend that females chose extra‐pair partners with more elaborate sexually selected signals, but the trend was significant only for song traits (Hsu et al. 2015); Hsu et al. (2015) found clear evidence for extra‐pair males being significantly older than cuckolded males. However, this pattern is not necessarily the consequence of female choice, and alternative, nonadaptive explanations for female infidelity have emerged (Forstmeier et al. 2014; Westneat and Stewart 2003). According to these hypotheses, female promiscuity is the mere result of the close genetic correlation between male and female propensity for promiscuity (“intersexual pleiotropy” hypothesis), or between female promiscuity and other female traits, such as female fecundity or responsiveness towards the social male (“intrasexual pleiotropy” hypothesis) (Forstmeier et al. 2014).

Regardless of the underlying mechanism of promiscuity, some costs of promiscuous behaviour are expected to appear, and identifying these is crucial in understanding of the evolution of extra‐pair mating behaviour. First of all, engaging in EPCs may imply search costs for both sexes (Dunn and Whittingham 2007), but it may also elevate the risk of encountering sexually transmitted diseases (Poiani and Wilks 2000) that can lead to health deterioration and decreased fertility (Sheldon 1993). Furthermore, males seeking additional mating opportunities may face other males' aggression, or risk losing their paternity in the broods laid by their social partners, if there is a trade‐off between mate guarding (known to increase within‐brood paternity; Harts et al. 2016) and extra‐pair mating (as suggested by Johnsen and Lifjeld 2003, Eikenaar 2008). In the case of females, additional costs may arise if their social mates retaliate their infidelity. Male's retaliation may manifest in aggression against the unfaithful social partner (Valera et al. 2003) or decreased parental investment in the brood laid by the social partner (Møller and Cuervo 2000; Søraker et al. 2023).

According to the certainty of paternity hypothesis (as proposed in Møller 1988), cuckolded males reduce their parental investment in response to their social mate's infidelity, because any investment in unrelated offspring entails costs without any fitness benefits. Such adjustment requires that the males are able to assess their paternity (Westneat and Sherman 1993). Although males are probably unable to identify their genetic offspring (Kempenaers and Sheldon 1996), they may be able to assess the certainty of their paternity by indirect cues, such as their mate's behaviour (e.g., leaving the territory or being away from the male for prolonged time, soliciting copulation from or copulating with foreigner males) or the interest of neighbouring males in their mate during the fertile period (Sheldon 2002). In line with the hypothesis, some studies demonstrated a negative link between males' parental care and their genetic paternity in the broods laid by their social partners (e.g., nest defence: Lubjuhn et al. 1993 and Weatherhead et al. 1994; nestling provisioning: Dixon et al. 1994; Chuang‐Dobbs et al. 2001; Suter et al. 2009 and Schroeder et al. 2016) or their experimentally manipulated certainty of paternity (e.g., nest provisioning: Møller 1988; Lifjeld et al. 1998 and Sheldon and Ellegren 1998) (for an earlier review see Whittingham and Dunn 2001). But in addition to the supportive studies, there are others that have not found evidence in favour of the certainty of paternity hypothesis (e.g., Hoi et al. 2013; MacDougall‐Shackleton and Robertson 1998; Peterson et al. 2001; Poblete et al. 2021; Yezerinac et al. 1996).

Inconsistent results regarding the certainty of the paternity hypothesis were found also between populations of the same species (see Li et al. 2021 and references therein). This is not surprising, because a couple of species‐ or population‐specific characteristics were suggested to influence the relationship between paternity and paternal care. For example, the meta‐analysis of Griffin et al. (2013) showed that the expected relationship is likely to appear if the costs of paternal care (in terms of survival) and the rate of EPP in the population are both high. The model of Mauck et al. (1999), on the other hand, suggested that from the point of the appearance of reduced care, the accuracy of the male's information on paternity may be more important than the rate of EPP per se. In real life, however, these two are likely to be interconnected, because while seeking EPCs males are probably not able to observe the behaviour of their social partners (Magrath and Komdeur 2003) and thus reliably assess their paternity in their own broods. Therefore, in populations where the majority of the males are seeking extra‐pair mating opportunities, we may expect that the relationship between males' certainty of paternity and parental investment is weak or absent, while in populations with a moderate rate of EPP, the relationship may be significant.

Earlier studies (Sheldon et al. 1997; Sheldon and Ellegren 1998) found reduced feeding effort in response to the males' uncertainty of paternity in a Swedish collared flycatcher ( *Ficedula albicollis* ) population with moderate EPP rate (33% of the broods contained EPY; Sheldon and Ellegren 1999). In our study population, the incidence of EPP is much higher, with 56% of broods containing EPY (Rosivall et al. 2009). This rate can be considered high in birds with biparental care, as only the minority (15%) of the surveyed species had a population with an EPP rate higher than 55% (calculated from Table S1 of Brouwer and Griffith (2019)). Our aim was to test the certainty of paternity hypothesis in this population with high rate of EPP using a cross‐fostering design. In line with our prediction in the previous paragraph, we expected that the relationship between male's certainty of paternity and parental investment is weak or absent.

Cross‐fostering design was used for multiple reasons. Although offspring recognition is unlikely in monogamous or noncolonial bird species (e.g., *Prunella modularis* : Davies et al. (1992), *Sialia mexicana* : Leonard et al. (1995), *Passer domesticus* : Schroeder et al. (2016); Lattore et al. (2019)) and extra‐pair nestlings do not differ in any traits from within‐pair nestlings in the Central European populations of our study species (Krist and Munclinger 2011; Rosivall et al. 2009; Rosivall et al. 2010; Wilk et al. 2008), if begging intensity depends on the relatedness of the competing nestlings (Boncoraglio et al. 2009), nestlings in mixed paternity broods and in broods of faithful females may beg with different intensity, and this may cause differences in the feeding rate of the males even if they are unable to evaluate their paternity. In addition, it is known that female quality may influence feeding rates via the quality of the offspring (Kiss et al. 2013) and females that engage in EPCs and those that do not may differ in quality (Rosivall et al. 2009), possibly leading to a correlation between paternity and feeding rate without males being able to evaluate their paternity. Using a cross‐fostering design, where all offspring were unrelated to the foster parents and came from two different broods, the above‐mentioned problems were eliminated, and we ensured that males could assess the certainty of their paternity only through indirect cues (e.g., female mating behaviour during the fertile period) and not through direct cues (e.g., offspring behaviour).

## 2 Methods

### 2.1 Study Site and Population

The study was conducted in a collared flycatcher population breeding at an artificial nest box settlement located in a protected area in the Pilis‐Visegrád Mountains, Hungary (47°43′ N, 19°01′ E). The plots used in this study covered approximately 33 ha in an oak‐dominated forest. The population of this insectivorous passerine species spends the winter in Sub‐Saharan Africa and returns to its breeding area in the middle of April. Although they are primarily socially monogamous, polygyny occurs in the population (Herényi et al. 2012). Pair bonds usually persist for one breeding event, and normally females lay one clutch per breeding season. During the incubation period, only females incubate their typically five to seven eggs, but males provide supplemental food for their mates (Kötél et al. 2016). During the offspring rearing period, both parents feed their nestlings. Hatching asynchrony is common, as females often start the incubation before clutch completion. Most nestlings fledge when they are 15–16 days old.

### 2.2 Field Methods and Experimental Design

**FIGURE 1:**
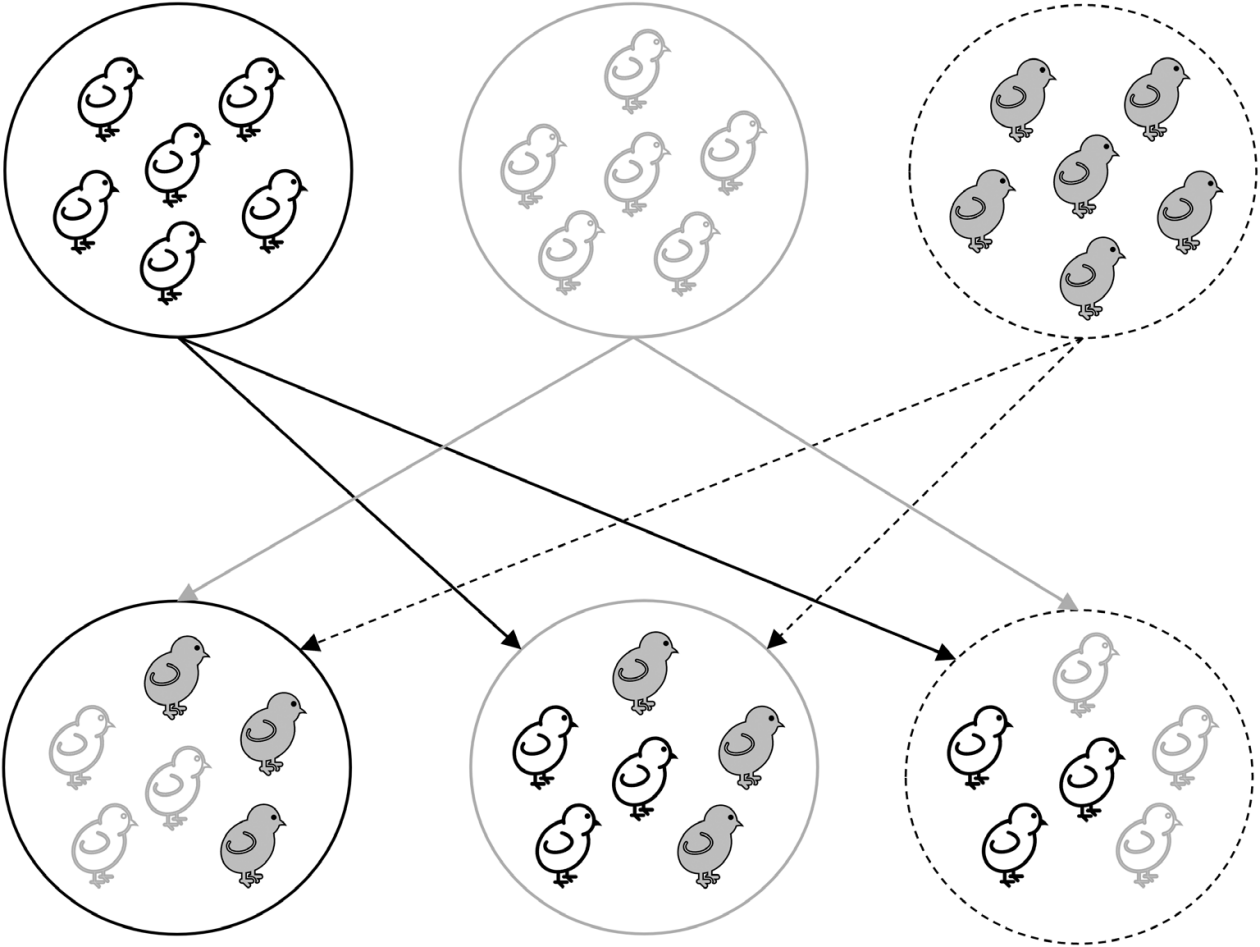
The experimental design. The circles symbolise the broods, and the arrows indicate the direction of swapping, with the upper part representing the stages before and the lower part representing the stages after the cross‐fostering.

During the breeding seasons of 2017 and 2018, we cross‐fostered nestlings between trios of nests 2 days after the hatching of the first nestling of the brood (we had 15 trios 10 in 2018). The three broods of a trio shared the same hatching date and were either identical in brood size or the difference was not more than one offspring. After the cross‐fostering, all parents got the same number of nestlings as originally hatched; thus, brood size was not manipulated. All nestlings were unrelated to the foster parents, and they came from two different broods approximately in an equal proportion (Figure 1). We chose this design because, in the case of cross‐fostering full broods between duos of nests, some broods would have contained only full sibs, while others would have contained half‐sibs, and the begging behaviour of the offspring could have differed between the two types of broods (see introduction). This could have introduced large random noise and reduced statistical power. Note that all males included in our analyses raised unrelated nestlings only; thus, even if male feeding rate was influenced by the experiment, all individuals would have been affected uniformly. However, the results of Schroeder et al. (2016) suggest that cross‐fostering is not likely to affect male feeding rates or the relationship between male feeding rate and paternity.

During cross‐fostering, offspring were marked individually by removing tufts of down on their head and back in unique combinations and were weighed to the nearest 0.1 g with a Pesola spring balance. Offspring were ringed on the sixth day posthatching, and 2 days later a small blood sample was taken from each nestling by brachial vein puncture. Unhatched eggs and tissue samples from dead offspring were also collected. Parents were captured, ringed, measured, and blood sampled when nestlings were 9–11 days old.

### 2.3 Videotaping and Video Analysis

We monitored parental feeding activity using compact digital FHD video cameras during the peak of the feeding period, either on day 8 or day 9 after hatching, with one exception, where video was recorded when nestlings were 10 days old. We recorded the videos between 7 am and 2 pm using video cameras that were set up on a tripod ca. 10–15 m away from the nest boxes. Video recordings were later analysed using Media Player Classic Home Cinema (MPC‐HC, version 1.7.13). Behavioural data were recorded and analysed blindly with respect to the occurrence of EPP in the nest to minimise observer bias. As collared flycatchers are sexually dimorphic, males and females are easy to distinguish by feather colouration, so male and female feeding rates (number of feedings per hour) were calculated separately. We defined feeding events as the bird going into the nest box or leaning in the nest box from the hole, then leaving with an empty beak. Although parents were probably minimally influenced by the human disturbance (as they resumed their feeding behaviour just minutes after the recordings were initiated), from each video we discarded the minutes (median = 4.5 min) recorded before both parents resumed feeding. Furthermore, video length and number of feeding events were also corrected for the duration of accidental disturbance caused by humans (e.g., louder footsteps or talking in the surrounding area) or potential nest predators (e.g., woodpecker activities) by excluding the time of the disturbance until both parents resumed feeding the nestlings again. The net length of the video recordings (i.e., length after correction) was 95.86 ± 20.12 min (mean ± SD; range = 60.20–141.82), which observation length was suggested to provide an accurate estimate of provisioning rates in different passerines (Lendvai et al. 2015 and references therein).

### 2.4 Paternity Analysis

Blood and tissue samples collected in the field were preserved in absolute ethanol at −20°C, and DNA was extracted with an ammonium‐acetate method (Nicholls et al. 2000). For paternity assessment, we used eight polymorphic microsatellite loci (Cuμ4, FhU2, FhU4, Fhy405, Fhy407, Fhy428, Fhy431, Fhy452; Ellegren 1992; Primmer et al. 1996; Griffith et al. 1999; Gibbs et al. 1999; Leder et al. 2008).

Polymerase chain reactions (PCRs) were run using the Type‐it microsatellite PCR kit (QIAGEN), and the following thermal profile: 95°C for 5 min, 30 cycles of 95°C for 30 s, 56°C for 30 s, 72°C for 30 s and 60°C for 30 min. PCR products were analysed on an ABI 3130xl Genetic Analyzer (Applied Biosystems, Foster City, CA, USA) with a 50 cm capillary and POP‐7 polymer (Applied Biosystems, Foster City, CA, USA) using the internal size standard GeneScan 600 LIZ (Applied Biosystems, Foster City, CA, USA). Due to logistic constraints, two different softwares were used to determine fragment lengths: Thermo Fisher Scientific's online Peak Scanner Software for samples from 2017 (*N* = 27 broods) and Genemapper ver. 4.1 (Applied Biosystems, Foster City, CA, USA) for samples from 2018 (*N* = 17 broods). According to the parallel analyses of the same samples (5 samples on 8 loci), the maximum difference in the results was 0.09 base pair.

According to the analysis of population‐level data from two consecutive years (2018–2019; 552 adult individuals; our unpublished result) the combined nonexclusion probability (for a candidate male given the genotype of the female) for the 8 loci was 4.37 × 10^−6^, and all loci were in Hardy–Weinberg equilibrium (calculated by CERVUS 3.0.7, Kalinowski et al. 2007). The alleles of the offspring and their original parents (so not the ones that reared the offspring after the cross‐fostering) were compared. Assuming Mendelian inheritance, offspring with known mothers were classified as extra‐pair young if their genotypes did not match their putative father at two or more loci. No double‐locus mismatch was found between mothers and nestlings, indicating that mutations and nonamplifying alleles were unlikely to confound our results.

### 2.5 Statistical Analysis

Due to logistic constraints, not all of the experimental broods were videotaped. We included 44 broods (27 in 2017 and 17 broods in 2018) in our statistical analyses, where both parents fed the nestlings and were blood sampled. In all broods included in the analyses, the clutch size, as well as the brood size at the time of the video recording was known, and the male was monogamous (*N* = 39) or the observed brood was the primary brood of a polygynous male (*N* = 5). Pseudoreplication was not an issue in our analysis, as all parents were video‐recorded only once.

First, we examined whether the age of the offspring at the time of recording and the start of the recording within days were related to feeding rates because the values of these factors varied between nests for logistic reasons. We combined the single brood recorded on day 10 posthatch with the broods recorded on day 9. We found no effect of nestlings' age during the video recording (8 vs. 9/10 days) on male feeding rate (Mann Whitney U test: U = 103.00, Z = 0.850, *p* = 0.309). Due to the limited number of cameras, we video‐recorded broods in two sessions during the days. Consequently, the starting times of the videos showed a bimodal distribution. Whether video recordings were started before or after 10 am (i.e., the nests were recorded during the first or second recording session of the day) did not affect the feeding activity of the males either (t test: t = 0.205, *p* = 0.839). Consequently, we did not control for these factors in the statistical analyses.

We constructed two general linear models to investigate the relationship between the feeding rate of the male and the infidelity of its partner (the latter was estimated by the paternity of the male in their original brood). The models differed only in the variable used to quantify extra‐pair mating behaviour of the female. In one model, we defined infidelity as a continuous variable (i.e., proportion of EPY in the broods), while in the other one as a binary variable (i.e., presence of EPY in the broods). We defined infidelity in two ways because we are unaware of whether males respond (1) to the temporary absence of the female during clutch formation, which may be more properly captured by a binary variable, or (2) to the number of copulations with the female, which may influence fertilisation success (Török et al. 2003) and may be more properly captured by the proportion of EPY. The feeding rate of the males can potentially be affected by the feeding rate of the partner (Harrison et al. 2009), by the number of offspring they feed (Kiss et al. 2013; Krist 2009), and may vary between years, for example, as a consequence of the differences in food availability (e.g., Senécal et al. 2021). Thus, aside from the variables describing infidelity, the full models contained the study years (categorical variable), the female feeding rate (continuous variable) and the brood size at the time of video recording as background variables. Brood size varied between 5 and 7, but was 5 in only five broods. Therefore, we combined brood sizes 5 and 6, and used brood size as a two‐level categorical variable (brood size five and six vs. brood size seven). We checked the independence of predictor variables by variance inflation factors (VIF) and multicollinearity was not an issue (all VIF values < 1.24; function vif in car package, Fox and Weisberg 2019).

In the case of two embryos and one nestling, the alleles could not be unambiguously identified at some loci despite repeated laboratory analyses of their samples. The three broods containing these samples were not included in the statistical analyses regarding the proportion of EPY, as not all nestlings' genotypes from the broods were known. But these broods were used in the analyses regarding the presence of EPY in the broods because all three broods contained EPY among the siblings of the unsuccessfully genotyped individuals. Thus, we included 41 broods in the model regarding the proportion of EPY and 44 broods in the model regarding the presence of EPY.

We performed backward stepwise model simplification after running the full model. We eliminated nonsignificant (*p* > 0.05) terms step by step from the model, starting with the terms with the highest *p* value. Model residuals were normally distributed (Kolmogorov–Smirnov test: all *p* > 0.20) in each analysis of full and final models. For more reliable results, we re‐entered nonsignificant variables to the final model one by one (Hegyi and Laczi 2015) and presented the results accordingly (function Anova in car package, Fox and Weisberg 2019).

In five broods, one nestling died between hatching and the time of video recording. To be sure that mortality does not influence the results, we ran all analyses using two datasets: one with the full dataset and one without the broods with mortality. We also had five broods that were the primary broods of monogamous males. Neither the feeding rates nor the proportion of cuckolded males differed between monogamous and polygynous males (feeding rate (mean ± SD): monogamous males = 24.64 ± 7.29; polygynous males: mean ± SD = 23.02 ± 4.21; t test: t = −0.486, *p* = 0.293; proportion cuckolded: monogamous males: 26/39; polygynous males: 2/5). However, to verify that our results are not confounded by using data from the primary nest of polygynous males and broods with mortality, the two full models (one analysing the effect of the presence/absence, and one the proportion of EPY in the broods, see above) were run on four overlapping datasets: (1) on the whole dataset (i.e., also consisting of broods with mortality and primary nests of polygynous males), (2) on a dataset without the broods with mortality, (3) on a dataset consisting of monogamous males only and (4) on a dataset without broods with mortality and without broods of polygynous males.

For the statistical analyses, we used R 4.2.2 (R Core Team 2022) and STATISTICA 6.1 (StatSoft Inc. 2003).

## 3 Results

Altogether, 63.64% of the broods (28 from 44) contained extra‐pair young in the 2 years. Of the 278 nestlings (168 individuals from 2017 and 110 from 2018) successfully analysed, 64 (23.02%) were sired by extra‐pair males. When only the nestlings from the mixed paternity broods were considered, on average 35% of them were sired by extra‐pair fathers (the proportion ranged from 14% to 71%).

**FIGURE 2:**
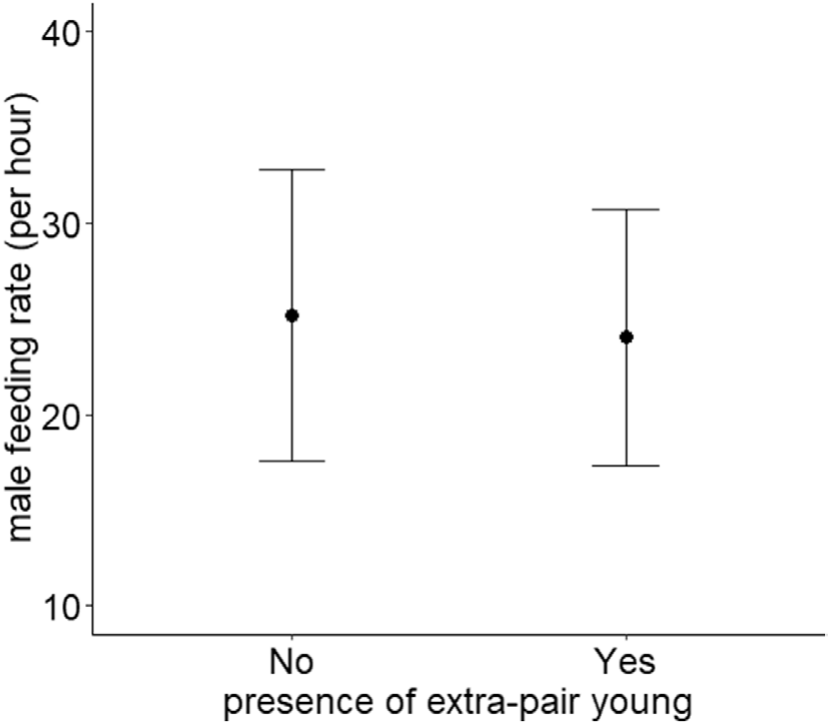
Paternal feeding rate and the presence of extra‐pair young. Means +/− standard deviations are shown.

**FIGURE 3:**
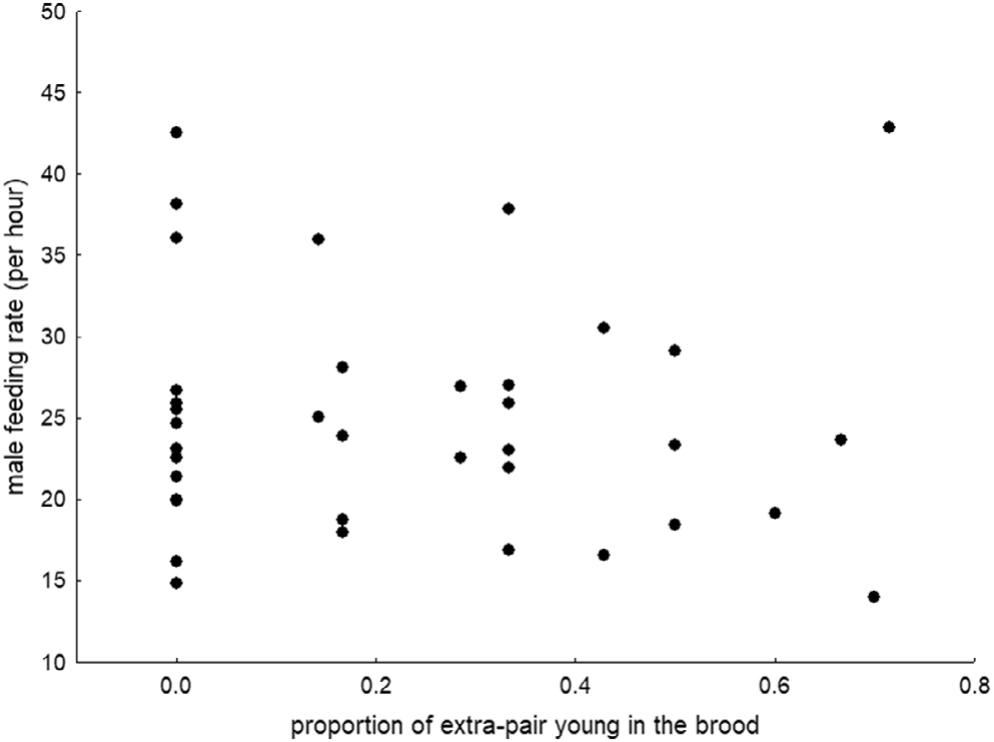
Paternal feeding rate and the proportion of extra‐pair young in the brood.

**FIGURE 4:**
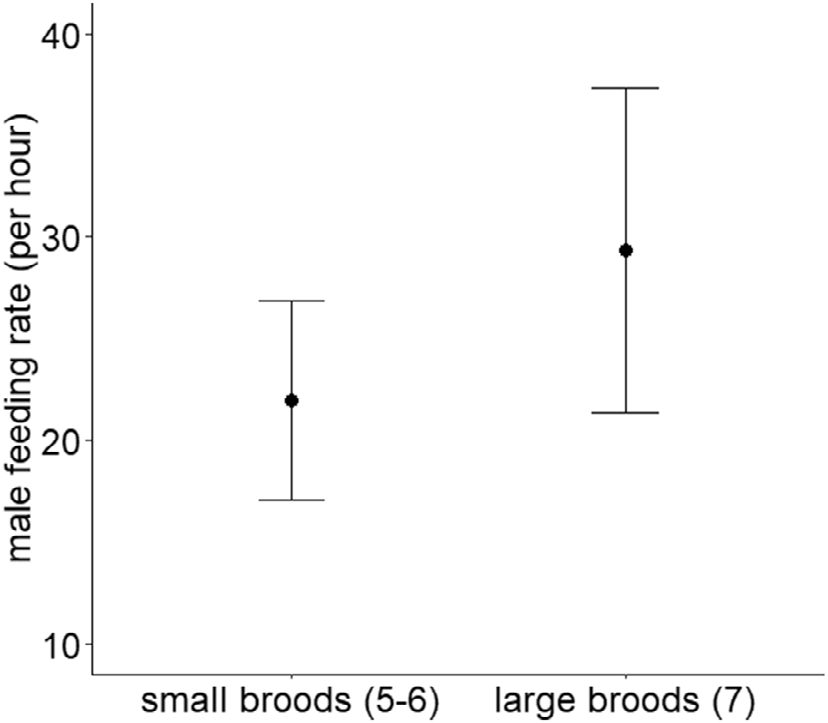
The relationship between paternal feeding rate and the brood size at the time of video recording. Means ± standard deviations are shown.

When we analysed the relationship between male feeding rates and their relatedness to their original nestlings, neither the presence of EPY (Table 1a, Figure 2) nor the proportion of EPY in the broods influenced paternal feeding rate (Table 1b, Figure 3). Furthermore, there was no relationship between male and female feeding rates (Table 1). Only the brood size at the time of video recording was associated with paternal feeding rate (Table 1), as males fed the nestlings more frequently in larger broods than in smaller broods (Figure 4).

**TABLE 1:** The effect of female infidelity on paternal feeding rate. The proxy of infidelity was either the presence/absence of EPY in the broods (a) or the proportion of EPY in the brood (b) (*N* = 44 and *N* = 41 respectively).

		Male feeding rate		
		df	*F*	*p*
(a)				
	Presence of EPY	1,41	0.13	0.719
	Brood size	**1,42**	**14.43**	**0.001**
	Partner feeding rate	1,41	0.002	0.968
	Year	1,41	1.20	0.280

(b)				
	Proportion of EPY	1,38	0.26	0.613
	Brood size	**1,39**	**12.22**	**0.001**
	Partner feeding rate	1,38	< 0.001	0.987
	Year	1,38	0.93	0.342

*Note:* Variables retained in the final model are highlighted in bold. F and *p* values for nonsignificant terms are obtained from the model containing the respective term and the term retained in the final model.

The trends were similar in the restricted datasets: neither the exclusion of broods where offspring mortality occurred before the time of video recording nor the exclusion of the primary broods of polygynous males nor the combination of these two exclusions changed the results qualitatively (seeS Appendix Tables A1, A2 and A3).

## 4 Discussion

According to our results, male flycatchers did not reduce their feeding rates in relation to their partners' infidelity, either in terms of the presence or the proportion of extra‐pair young in the brood. Our study adds to the increasing number of findings that do not support the certainty of paternity hypothesis (correlative studies: e.g., Westneat et al. 1995; Rytkönen et al. 2007; García‐Vigón et al. 2009; Schnitzer et al. 2014; Li et al. 2021; Poblete et al. 2021; Arrieta et al. 2022; experimental studies: e.g., Kempenaers et al. 1998; MacDougall‐Shackleton and Robertson 1998; Dickinson 1997; Hoi et al. 2013; Arrieta et al. 2022). On the other hand, there are findings, including experimental studies, that are in line with the hypothesis (correlative studies: e.g., Dixon et al. 1994; Chuang‐Dobbs et al. 2001; Suter et al. 2009; experimental studies: e.g., Møller 1988; Sheldon et al. 1997; Lifjeld et al. 1998; Sheldon and Ellegren 1998). Although a negative correlation between female infidelity and paternal investment may arise from various mechanisms that are not related to male retaliation (Schroeder et al. 2016), this cannot explain the discrepancies among the results of experimental studies in which paternity was manipulated.

The ability of the males to assess their paternity is a precondition of the certainty of paternity hypothesis. Therefore, variation in the relationship between infidelity and paternal care among species, or even populations of the same species, may be partly explained by variation in the accuracy of information on paternity (as suggested e.g., by Mauck et al. 1999). Males that are busy engaging in EPCs instead of guarding their mates may not only lose paternity (Harts et al. 2016), but may also lack information about the movements of their social partners, so they may be unable to reliably assess their paternity (Magrath and Komdeur 2003). Indeed, Schroeder et al. (2016) found that the relationship between within‐brood paternity (i.e., paternity in the brood of the social partner) and paternal care was influenced by the male's extra‐pair mating activity. Differences in extra‐pair mating activity may perhaps explain why our results contradict those found in a Swedish population of collared flycatchers (Sheldon et al. 1997; Sheldon and Ellegren 1998). In their studies, Sheldon and his colleagues observed reduced feeding rates of males with experimentally decreased certainty of paternity compared to control males (Sheldon et al. 1997; Sheldon and Ellegren 1998). In the Swedish population, where the prevalence of EPP is moderate (33%; Sheldon and Ellegren 1999) males may be able to assess their paternity relatively accurately, thus the adjustment of paternal care to the certainty of paternity may have evolved. On the other hand, in our study population with a high rate of infidelity (56% and 64%; Rosivall et al. 2009 and this study respectively), males may be unable to reliably assess their paternity. It is worth mentioning that if it is a general pattern that retaliation is not found in populations with high levels of EPP, it would be hard to decide whether the high EPP rate is the reason why there is no retaliation, or the lack of retaliation is the reason why the EPP rate is high.

Population differences in the relationship between paternity and paternal provisioning were also found in reed buntings ( *Emberiza schoeniclus* ). Similar to collared flycatchers, reed buntings are primarily socially monogamous passerines characterised by biparental care. EPCs are frequent in this species, and males guard their social partners during the fertile period (for more details see in Bouwman et al. 2005). While the results found in an English population (Dixon et al. 1994; *N* = 13) and a Swiss population (Suter et al. 2009; *N* = 14) supported the certainty of paternity hypothesis, no such evidence was found in a Dutch population (Bouwman et al. 2005; *N* = 10). In this case, the dissimilar results cannot be explained by the difference in the EPP rates. In the Swiss population, where a negative relationship between the proportion of EPY in a brood and a male's feeding rate was found, the rate of EPP was 64% (Suter et al. 2009), that is, similarly high as in our population, while in the English and the Dutch populations, where the EPP rate was even higher and quite similar (86% vs. 80% of broods contained EPY; Dixon et al. 1994; Bouwman et al. 2005 respectively), the results were contradictory. As Suter et al. (2009) argued, in this species, population differences in the results may be caused by differences in food availability and thus in the cost of parental care.

Not only may the accuracy of information on infidelity influence whether the relationship predicted by the certainty of paternity hypothesis appears in a population. In species where cuckolded males sire a considerable proportion of the offspring in their own brood, not adjusting paternal investment to the infidelity of the partner may be a reasonable strategy even if males can evaluate whether their partner was faithful. This is because the reduced fitness of the males' own (genetic) nestlings resulting from reduced paternal investment may entail larger costs than the cost of rearing unrelated offspring (Whittingham and Lifjeld 1995). If cuckolded males were to reduce their feeding effort in our study population, the likelihood of putting their own nestlings at a disadvantage would indeed be high, since on average, the majority of the nestlings (65%) in mixed paternity broods were the genetic offspring of the cuckolded males.

If some individual traits or ecological factors mask the effect of paternity on paternal investment, it may also explain the lack of the expected relationship. For example, it may be possible that under good conditions, males of faithful females feed the nestlings more frequently than cuckolded males, but under poor conditions, this difference is not apparent due to limited availability of food. Alternatively, aggressive males are more successful in guarding their females (Moreno et al. 2010), but during the nestling stage, they may invest less in parental care (Mutzel et al. 2013) than those males that guard their females less actively, probably due to the contrasting effects of testosterone on these behaviours (Silverin 1980). However, these explanations are unlikely because in the study years food abundance was not low (personal observation), and in this population, feeding behaviour was independent of the aggressiveness of the males (Szász et al. 2019).

The lack of the relationship between paternity and paternal care could also be explained by the differential allocation hypothesis, which suggests that a low‐quality individual should allocate greater investment in their current reproductive event in order to obtain and/or maintain a pair‐bond with a mate of higher quality (relative to itself) (Burley 1981, 1986). If females mated with poorer quality mates are more prone to engage in EPCs in this population (as suggested by Michl et al. 2002, but see Rosivall et al. 2009), then the benefits of obtaining and/or maintaining a pair‐bond with a higher quality female may counterbalance the losses caused by investment in some unrelated offspring, especially if the proportion of the offspring sired by the male is relatively high, as in our population (see above). In such cases, it may not be beneficial to adjust paternal care to infidelity.

Lastly, we cannot exclude the possibility that cuckolded males, in fact, decreased their parental investment, but (1) reduced other aspects of care (e.g., nest guarding) rather than nestling feeding, or (2) altered the quality, not the quantity, of the delivered food. Unfortunately, we did not have the opportunity to investigate other types of paternal investment or the food quality (e.g., prey type, food size) during the feeding events. However, Cauchard and her colleagues (2021) have concluded in their recent study on a collared flycatcher population that feeding rate was a better proxy for the total biomass delivered than prey size or mean number of prey per visit.

Concerning the background variables we included in our models, male and female feeding rate were independent of each other (in line with the results of Kiss et al. 2013), while paternal feeding rate was positively correlated with the size of the brood at the time of video recording. Higher feeding activity with more nestlings can be considered a general pattern, as parents are likely to react to offspring's begging calls that indicate the demands of the nestlings (Grieco 2001 and references therein). Previous results on collared flycatchers also suggest that parents adjust their feeding activity to the number of nestlings (Cauchard et al. 2021; Kiss et al. 2013; Krist 2009).

To summarise our findings, the certainty of paternity hypothesis is not supported in our study population, as males did not reduce their feeding rate in relation to the presence or proportion of the extra‐pair young. In other words, females did not suffer direct costs of infidelity in terms of reduced paternal care by their social mate. If there is a trade‐off for males between engaging in EPCs and assessing paternity in the broods laid by their social partners, the high rate of infidelity that we detected in the current study may explain the lack of the expected relationship. It may also explain why different results were found in another population of collared flycatchers. Further studies are clearly needed to clarify whether the rate of EPP may explain the variation among species and populations in the reaction of males to their partners' infidelity.

## Author Contributions

**Helga Gyarmathy:** data curation (lead), formal analysis (lead), investigation (equal), visualization (lead), writing – original draft (lead), writing – review and editing (lead). **Renáta Kopena:** data curation (supporting), investigation (equal), writing – review and editing (equal). **Tünde Kneifel:** data curation (supporting), investigation (equal), writing – review and editing (equal). **Fanni Sarkadi:** data curation (supporting), investigation (equal), writing – review and editing (equal). **Eszter Szöllősi:** funding acquisition (supporting), investigation (equal), methodology (equal), writing – review and editing (equal). **Eszter Szász:** investigation (equal), methodology (equal), writing – review and editing (equal). **János Török:** investigation (equal), writing – review and editing (equal). **Balázs Rosivall:** conceptualization (equal), data curation (supporting), formal analysis (supporting), funding acquisition (lead), investigation (equal), methodology (equal), supervision (lead), writing – review and editing (equal).

## Ethics Statement

Trapping, ringing and blood sampling of birds were conducted according to protocols established during the long‐term monitoring of the study population of collared flycatchers since the early 1980s. Adult birds were trapped with spring traps attached to the nest box entrance when the nestlings were already endothermic and did not require brooding. The birds were released immediately after measurements and resumed breeding activities soon thereafter. During the cross‐fostering, nestlings were transferred in soft cotton bags placed in boxes heated with reusable pocket warmer packs. The cross‐fostering apparently had no adverse effect on the nestlings because fledging success in the experimental nests was similar to that in unmanipulated nests. To reduce disturbance, we used external video cameras set up approx. 10–15 m away from the recorded nest box. The nestlings continued to receive food shortly after the start of the video recording (*X* ± SD = 120 ± 90 s, *N* = 44). The use of animals adheres to the guidelines set forth by the Animal Behaviour Society/Association for the Study of Animal Behaviour (2024). This study received prior approval from the National Scientific Ethical Committee on Animal Experimentation and the Department of Environment and Nature Protection of the Hungarian Government Office (case numbers PE/KTF/11978–6/2015, PEI/001/1054–6/2015, PE‐06/KTF/3331–4/2018, PE/EA/77–8/2018).

## Conflicts of Interest

The authors declare no conflicts of interest.

## Supporting information


Appendix S1.

## Data Availability

All data analysed in the current study are available in the repository of Eötvös Loránd University at https://edit.elte.hu/xmlui/handle/10831/115498.

## Appendix A

TABLE A1. The effect of female infidelity on paternal feeding rate according to the analyses run on a restricted dataset containing only those broods where the original clutch size was identical to the brood size at video recording. The proxy of infidelity was either the presence/absence of EPY in the broods (a), or the proportion of EPY in the brood (b) (*N* = 39 and *N* = 37 respectively).

**Table:**

		Male feeding rate		
		df	*F*	*p*
(a)				
	Presence of EPY	1,36	0.22	0.642
	Brood size	**1,37**	**12.08**	**0.001**
	Partner feeding rate	1,36	< 0.001	0.993
	Year	1,36	1.01	0.321
(b)				
	Proportion of EPY	1,34	0.28	0.598
	Brood size	**1,35**	**10.55**	**0.003**
	Partner feeding rate	1,34	< 0.001	0.986
	Year	1,34	0.84	0.367

*Note:* Variables retained in the final model are highlighted in bold. *F* and *p* values for nonsignificant terms are obtained from the model containing the respective term and the term retained in the final model.

TABLE A2. The effect of female infidelity on paternal feeding rate according to the analyses run on a restricted dataset containing broods of monogamous males only. The proxy of infidelity was either the presence/absence of EPY in the broods (a), or the proportion of EPY in the brood (b) (*N* = 39 and *N* = 36 respectively).

**Table:**

		Male feeding rate		
		df	*F*	*p*
(a)				
	Presence of EPY	1,36	0.53	0.472
	Brood size	**1,37**	**21.12**	**< 0.001**
	Partner feeding rate	1,36	0.011	0.919
	Year	1,36	1.48	0.233
(b)				
	Proportion of EPY	1,33	1.13	0.295
	Brood size	**1,34**	**17.90**	**< 0.001**
	Partner feeding rate	1,33	0.029	0.867
	Year	1,33	1.18	0.285

*Note:* Variables retained in the final model are highlighted in bold. *F* and *p* values for nonsignificant terms are obtained from the model containing the respective term and the term retained in the final model.

TABLE A3. The effect of female infidelity on paternal feeding rate according to the analyses run on a restricted dataset containing only broods of monogamous males where the original clutch size was identical to the brood size at video recording. The proxy of infidelity was either the presence/absence of EPY in the broods (a), or the proportion of EPY in the brood (b) (*N* = 34 and *N* = 32 respectively).

**Table:**

		Male feeding rate		
		df	*F*	*p*
(a)				
	Presence of EPY	1,31	0.80	0.378
	Brood size	**1,32**	**17.64**	**< 0.001**
	Partner feeding rate	1,31	0.02	0.898
	Year	1,31	1.24	0.275
(b)				
	Proportion of EPY	1,29	1.34	0.256
	Brood size	**1,30**	**15.39**	**< 0.001**
	Partner feeding rate	1,29	0.02	0.881
	Year	1,29	1.05	0.314

*Note:* Variables retained in the final model are highlighted in bold. *F* and *p* values for nonsignificant terms are obtained from the model containing the respective term and the term retained in the final model.
